# Dopamine Transporter Neuroimaging as an Enrichment Biomarker in Early Parkinson's Disease Clinical Trials: A Disease Progression Modeling Analysis

**DOI:** 10.1111/cts.12492

**Published:** 2017-07-27

**Authors:** Daniela J. Conrado, Timothy Nicholas, Kuenhi Tsai, Sreeraj Macha, Vikram Sinha, Julie Stone, Brian Corrigan, Massimo Bani, Pierandrea Muglia, Ian A. Watson, Volker D. Kern, Elena Sheveleva, Kenneth Marek, Diane T. Stephenson, Klaus Romero, on behalf of the Critical Path for Parkinson's (CPP) Parkinson's Disease Modeling and Simulation Working Group

**Affiliations:** ^1^ Critical Path Institute Tucson Arizona USA; ^2^ Pfizer Inc Groton Connecticut USA; ^3^ Merck Sharp & Dohme North Wales Pennsylvania USA; ^4^ UCB Brussels Belgium; ^5^ Eli Lilly Indianapolis Indiana USA; ^6^ University of Arizona Tucson Arizona USA; ^7^ Institute for Neurodegenerative Disorders New Haven Connecticut USA

## Abstract

Given the recognition that disease‐modifying therapies should focus on earlier Parkinson's disease stages, trial enrollment based purely on clinical criteria poses significant challenges. The goal herein was to determine the utility of dopamine transporter neuroimaging as an enrichment biomarker in early motor Parkinson's disease clinical trials. Patient‐level longitudinal data of 672 subjects with early‐stage Parkinson's disease in the Parkinson's Progression Markers Initiative (PPMI) observational study and the Parkinson Research Examination of CEP‐1347 Trial (PRECEPT) clinical trial were utilized in a linear mixed‐effects model analysis. The rate of worsening in the motor scores between subjects with or without a scan without evidence of dopamine transporter deficit was different both statistically and clinically. The average difference in the change from baseline of motor scores at 24 months between biomarker statuses was –3.16 (90% confidence interval [CI] = –0.96 to –5.42) points. Dopamine transporter imaging could identify subjects with a steeper worsening of the motor scores, allowing trial enrichment and 24% reduction of sample size.

**Study Highlights**

**WHAT IS THE CURRENT KNOWLEDGE ON THE TOPIC?**

✓ Disease‐modifying therapies are likely to provide benefit in earlier PD. However, trial enrollment based purely on clinical criteria poses significant challenges given the high heterogeneity of symptoms and pathophysiology in patients with early‐stage PD.

**WHAT QUESTION DID THIS STUDY ADDRESS?**

✓ It addressed the utility of DAT neuroimaging as an enrichment biomarker in clinical trials targeting patients with early‐stage PD.

**WHAT THIS STUDY ADDS TO OUR KNOWLEDGE**

✓ The rate of worsening in the motor scores between subjects with or without a scan without evidence of DAT deficit (SWEDD) was different both statistically and clinically. DAT imaging identified subjects with a steeper worsening of the motor scores, allowing trial enrichment and ∼24% reduction of trial size.

**HOW THIS MIGHT CHANGE CLINICAL PHARMACOLOGY OR TRANSLATIONAL SCIENCE**

✓ Exclusion of SWEDD subjects (DAT imaging‐based enrichment) from future clinical trials will improve the chance of determining clinical benefit of new drug candidates against PD at a reduced trial size, and prevent exposure to experimental treatments of patients who are unlikely to experience disease progression.

Parkinson's disease (PD) remains an unmet medical need,^1^, ^2^ affecting 1 million Americans, with at least 60,000 new cases being reported annually.^3^ Current therapies are symptomatic, which do not alter the underlying neurodegenerative process.^4^

Treating earlier may help demonstrate disease modification, but trial enrollment based purely on clinical criteria poses significant challenges. Heterogeneity of symptoms and pathophysiology is also high in subjects with early‐stage PD. Approximately 11% subjects without evidence of dopamine transporter (DAT) deficit (SWEDD) were enrolled in the failed Parkinson Research Examination of CEP‐1347 Trial (PRECEPT), presenting minimal clinical or imaging changes over time.^5^

Reduction of DAT density is more sensitive than clinical examination to detect nigrostriatal dopaminergic deficit. Reduction of DAT radiotracer binding, as assessed by single‐photon emission computed tomography neuroimaging, reflects dopaminergic nerve terminal degeneration in subjects with PD, which precedes the onset of clinical symptoms.^6^ DAT‐selective radioligand [^123^I]N‐ω‐fluoropropyl‐2β‐carbomethoxy‐3β‐[4‐iodophenyl]nortropane is the one currently approved for differential diagnosis between PD and essential tremor.^7^, ^8^ However, there is no current regulatory endorsement for the use of DAT imaging as an enrichment biomarker in clinical trials.

The goal of this effort was to determine the utility of DAT neuroimaging as an enrichment biomarker in clinical trials targeting early‐stage PD, of up to 24 months in duration. Confirming reduction of DAT density by single‐photon emission computed tomography in subjects with early motor deficit is proposed as a means of enriching future clinical trials of PD therapeutic agents as this facilitates excluding patients who are unlikely to show disease progression in a PD clinical trial. This work was carried out by Critical Path Institute's Critical Path for Parkinson's (CPP) Consortium (funded by Parkinson's United Kingdom).^9^ The CPP's focus is to create new tools and methods that can be applied during the development process of new treatments for PD. The CPP will achieve this by sharing knowledge and developing consensus on new tools that will be submitted to regulatory agencies for formal review and endorsement. This will also advance the field of regulatory science accelerating drug development for PD.

## METHODS

### Data

#### Population

The target population for use of DAT imaging as an enrichment biomarker is subjects recently diagnosed with PD who are treated with minimal to no dopaminergic medications and have early signs of motor symptoms. Criteria for early‐stage PD was: (a) baseline Hoehn and Yahr stage I or II; (b) two of the following signs: resting tremor, bradykinesia, and rigidity; or (c) either asymmetric resting tremor or asymmetric bradykinesia.^1^

#### Studies

Longitudinal subject‐level data were integrated from two large multicenter global clinical studies focusing on early‐stage PD: the PRECEPT^10^ and the Parkinson's Progression Markers Initiative (PPMI) observational study.^11^ PRECEPT was a phase II/phase III, multicenter, randomized, double‐blind, placebo‐controlled, dose‐finding trial to determine the efficacy and long‐term safety of CEP‐1347 (mixed lineage kinase inhibitor) as a potential disease‐modifying treatment in subjects with early PD. A total of 806 subjects were enrolled in the trial and the primary clinical end point was time to the development of disability requiring dopaminergic therapy. Planned treatment duration was a minimum of 24 months. Although the trial was stopped earlier for futility, after all subjects had been observed for an average of 21.4 months and 200 subjects for at least 24 months, the collected data on DAT imaging biomarker at baseline and during long‐term clinical follow‐up in the precise target population of interest represent a rich source for analyses. PPMI is an ongoing multicenter observational study supported by a consortium of academic centers, PD foundations, and pharmaceutical and biotechnology companies to collectively design, fund, and implement a comprehensive research program. The primary objective of the PPMI is to identify clinical, imaging, and biologic markers of PD progression for use in clinical trials of disease‐modifying therapies. Recruitment for PPMI began in 2010 and is ongoing for certain target groups.

#### Data standardization and inclusion/exclusion criteria

Considered within the scope of this analysis were (a) the PD cohort in PPMI; (b) the SWEDD cohort in PPMI; and (c) the placebo arm in PRECEPT. The treatment arm in PRECEPT was not used because an examination of drug effect was beyond the scope of this work. Conforming to comprehensive data standards was essential to the development of a database that enabled the pooling of data from different sources for integrated analyses. For this, the CPP used existing data standards published by the Clinical Data Interchange Standards Consortium (www.cdisc.org), a nonprofit organization that focuses on developing global standards for clinical trial data collection. These standards included the foundational Study Data Tabulation Model and the Therapeutic‐Area User Guide version 1.0 for PD (https://www.cdisc.org/standards/therapeutic-areas/parkinsons-disease).

Data excluded from the analysis were: (a) observations that occurred in time before baseline assessments (e.g., screening); (b) observations that occurred in time ≥25 months, based on the follow‐up time in the PRECEPT study (please refer to the Studies section); and (c) subjects with missing DAT biomarker status per visual interpretation. Subjects with at least one observation of the dependent variable were included in the analysis, and imputation of missing observations was not conducted before analysis.

#### Time metric and dependent variable

The time metric was the time in the study in months. The dependent variable was the harmonized Unified Parkinson's Disease Rating Scale (UPDRS) and Movement Disorder Society‐UPDRS (MDS‐UPDRS) part III score, referred to henceforth as the motor score. This metric was generated after two stages. For each individual observation: (1) the UPDRS or MDS‐UPDRS part III subitems were summed to generate the part III subtotal; and (2) the UPDRS part III subtotal was transformed to the respective MDS‐UPDRS part III to yield the harmonized motor score. The transformation of the individual UPDRS part III subtotal to the respective MDS‐UPDRS relied on a previously derived formula based on a Hoehn and Yahr stage I or II^12^:

(1) $$MDS-UPDRS_{III}=UPDRS_{III}\cdot 1.2+2.3$$

#### Dropout analysis

A dropout analysis was conducted within the baseline‐to‐25‐month interval to shed light on the missing data mechanism. Exploratory examination of Kaplan‐Meier curves was followed by parametric model‐fitting, including the following distributions: exponential; Weibull; log‐normal; gamma; log‐logistic; and Gompertz. Relevant subject characteristics were also tested for association with dropout. Model selection was guided by a modified version of the Akaike information criterion (AIC_mod_ 13) with a per‐parameter penalty of 3.841 to be equivalent to a one‐parameter, nested‐model  χ^2^ test with a *P* value of 0.05.

### Statistical model

The course of the harmonized motor scores over the months in the study was described using a linear mixed‐effects model, assuming a linear trajectory of the scores over time. Interindividual variability was allowed for baseline (i.e., intercept) and progression rate (i.e., slope) through the incorporation of random effects. Prespecified covariates were: (a) effect of biomarker status in baseline motor scores and progression rate; and (b) effect of the study in baseline motor scores to account for potential score differences between the PPMI and PRECEPT populations. The prespecified mixed‐effects model is represented in **Eq. S1** (**Supplementary Material**). Exploratory covariates were: (a) effect of age in baseline motor scores and progression rate given the neurodegenerative nature of PD; and (b) effect of the study in the progression rate to investigate the potential rate differences between the PPMI and PRECEPT. For comparison, a model without any adjustment for biomarker status was also fitted (i.e., reduced model).

The rate of progression on the motor scores was compared between SWEDD and DAT‐deficit subjects. The following null and alternative hypotheses were tested at one‐tailed α of 0.05:

- Null hypothesis (*H*_0_): The SWEDD progression rate is equal to or greater than that of DAT‐deficit subjects.
- Alternative hypothesis ($H_{a}$): The SWEDD progression rate is less than that of DAT‐deficit subjects.

Model selection criteria and evaluation of model performance are described in the **Supplementary Material**.

### Comparison of magnitude of motor scores worsening between biomarker statuses

The difference in the magnitude motor scores worsening between DAT‐deficit and SWEDD subjects was compared for clinical relevance. A previous cross‐sectional analysis identified the clinically important difference (CID) of 2.5 points for UPDRS part III, using a distribution‐based and an anchor‐based approach to data of 653 subjects diagnosed with PD who underwent routine UPDRS assessments during 41 months.^14^ Applying the conversion formula^12^ to translate such difference to the MDS‐UPDRS part III, the corresponding minimal CID equals 3 points.

Quantities of interest were calculated as following:

- The estimated mean progression rate for DAT‐deficit subjects, multiplied by 24, yielded the average magnitude motor scores worsening (change from baseline) at 24 months for this group.
- The estimated mean progression rate for SWEDDs, multiplied by 24, yielded the average magnitude motor scores worsening (change from baseline) at 24 months for this group.
- The estimated mean progression rate for DAT‐deficit subjects, subtracted from the mean progression rate for SWEDDs, and multiplied by 24, yielded the average difference in the magnitude motor scores worsening (change from baseline) at 24 months between biomarker statuses.

The 90% confidence intervals (CIs) for the above quantities (from the parametric bootstrap) were also multiplied by 24 months to yield the respective confidence ranges.

### Identification of subjects who experience a clinically important worsening of the motor scores

The combination of clinical criteria for early motor PD (see Data section) with DAT imaging vs. clinical criteria only was compared in terms of the ability to identify subjects who experienced a CID. The harmonized motor scores at baseline and 24 months were predicted for each subject. Subtracting the baseline from the 24‐month score yielded the individual change‐from‐baseline difference. The number of subjects with a difference ≥3 points (i.e., CID) was summarized for the analysis data set:

- The number of subjects with DAT deficit was calculated to yield the ability of the DAT imaging to identify patients who experienced a CID.
- The number of SWEDD subjects was calculated to yield the proportion of subjects who experienced a CID and would be excluded in a DAT‐based enriched trial enrolling only DAT‐deficit subjects.

### Clinical trial simulations and statistical power analysis

Monte Carlo‐based clinical trial simulations were performed to compare the statistical power vs. sample size in trials with and without DAT imaging enrichment. Enriched trials had only subjects with DAT deficit, whereas nonenriched trials included 15% of SWEDD subjects.^5^

Two thousand placebo‐controlled clinical trials with and without enrichment were simulated using the fixed and random effect parameter values from the chosen model, for a PRECEPT‐like study. The 24‐month trial sizes ranged from 100–700 subjects per arm. A hypothetical drug effect of 50% reduction in the disease progression rate was simulated for subjects with DAT deficit in the drug arms, assuming no effect on SWEDD subjects.

For each simulated trial, a linear mixed‐effects model was fitted and *P* values were calculated as described above (Generalized Linear Mixed‐Effects Model section). Fixed effects and random effects were as in the chosen model, except for the fixed effect of biomarker status and its interaction with time, which were not accounted for. The power, probability of detecting the drug effect, was calculated as the proportion of trials for which the parameter estimate for the interaction between time and treatment showed a beneficial drug effect and its two‐tailed *P* value was under 0.05.

## RESULTS

### Data summary

The analysis data set included a total of 672 subjects diagnosed with early‐stage PD and a total of 4,521 observations in the baseline‐to‐25‐month interval. Unscheduled visits with known time in the baseline‐to‐25‐month interval were also included. There were six subjects with missing biomarker status who were not included in the analysis data set (**Supplementary Figure S1**). Other exclusions occurred at the visit level and reasons are listed in **Supplementary Table S1**.

**Table** 1 shows the subjects’ baseline demographics and clinical characteristics stratified by the study. Subjects were between the ages of 31 and 84 years with a mean age of ∼60 years in both studies. Most subjects in each study were men with DAT deficit. The proportion of SWEDD subjects in the analysis data set was 13% and 14% for PPMI and PRECEPT, respectively. The mean harmonized motor scores at baseline of ∼20 points were similar for both studies.

**Table 1:** Baseline characteristics by study

**Baseline**	**PPMI**	**PRECEPT**
Sample size	481	191
Sex (%)	Female (35), male (65)	Female (34), male (66)
Age, years, mean (range)	61 (33–84)	59 (31–84)
DAT deficit (%)	Yes (87), no (13)	Yes (86), no (14)
Harmonized motor scores, mean (range)	20 (2–51)	21 (5.3–52)

DAT, dopamine transporter; PPMI, Parkinson's Progression Markers Initiative; PRECEPT, Parkinson Research Examination of CEP‐1347 Trial.

### Dropout model

**Figure 1:**
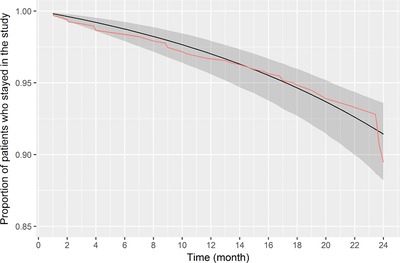
Dropout in Parkinson's Progression Markers Initiative (PPMI) and Parkinson Research Examination of CEP‐1347 Trial (PRECEPT) over time. The pink line corresponds to Kaplan‐Meier (nonparametric) estimates based on the observed data; the black line with shading corresponds to model predictions with the 95% confidence interval.

Dropouts in PPMI and PRECEPT represented 0.05 (95% CI = 0.03–0.07) and 0.09 (95% CI = 0.05–0.14], respectively, in the baseline‐25‐month interval. The Gompertz distribution was selected to describe the dropout pattern in both studies based on the AIC_mod_ (**Supplementary Table S2**). No statistically significant association between dropout and study, age, biomarker status, or baseline harmonized motor scores was found (**Supplementary Table S2**), with a trend of higher age for dropout subjects (62.79; 95% CI = 60.50–65.08 vs. 59.97; 95% CI = 57.77–62.18). The 95% CI of the model predictions captures the observed dropout (**Figure** 1), making a joint dropout and linear mixed‐effects models unnecessary.

### Linear mixed‐effects model

A linear mixed‐effects model, with a normal error distribution and an identity link function, was utilized to describe the trajectory of the harmonized motor scores over time and compare the rate of progression on the harmonized score between SWEDD and DAT‐deficit subjects. The full model includes all the prespecified parameters (**Eq. S1, Supplementary Material**) and all statistically significant exploratory covariates. In this case, age effect on the baseline motor scores was the only statistically significant exploratory covariate (two‐sided *P* value < 0.05). The prespecified study effect on the baseline motor scores seems to explain some of the variability, although not beyond the commonly used threshold (i.e., two‐sided *P* value of 0.05). The reduced model was as the full model, except for lacking the effect of biomarker status in baseline motor scores and disease progression rate. The R code, output summary, and analysis of variance table for reduced model, full model, and model comparison can be found in the **Supplementary Material**.

**Figure 2:**
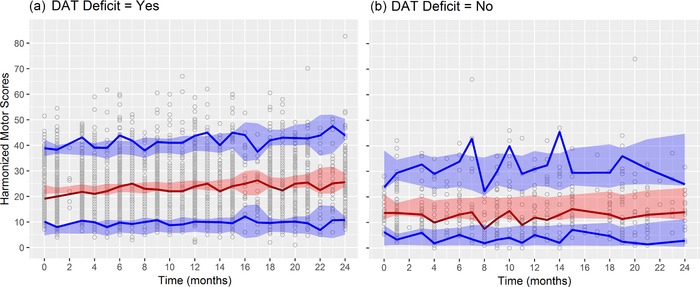
Visual predictive check for the full model. One thousand simulations were performed. The solid red line is the median of the observed scores; solid blue lines are the 5th and 95th percentiles of the observed scores; shaded areas are the 95% interpercentile ranges of the simulations. DAT, dopamine transporter.

Full model diagnostics suggest an adequate fit of the longitudinal changes in the harmonized score. The AIC for the reduced and full models was 29,713.22 and 29,637.17, respectively, indicating improvement when considering biomarker status. The **Supplementary Material** presents the comparison between models with additional statistics. For the full model, the individual‐observed vs. predicted motor scores approximated the identity line; Pearson residuals vs. individual‐predicted motor scores and time were mostly within ±3; and the assumption of normality held (**Supplementary Figure S2**). Pearson residuals vs. individual‐predicted motor scores presented some degree of heteroscedasticity. A sensitivity analysis was conducted by fitting the full model with the harmonized motor scores in the natural logarithm and logit domains. These transformations did not improve the heteroscedasticity, yielding increased Pearson residuals for the lower scores as compared with those for the higher scores. For illustration purposes, the first nine individuals (DAT‐deficit and SWEDD) observed and predicted harmonized motor scores (from the full model) vs. time graphs are presented in **Supplementary Figure S3**. Box plots on individual random effects for rate of progression stratified by DAT biomarker status for the reduced and full model are presented in **Supplementary Figure S4**. Unlike for the reduced model, the random effects for SWEDD subjects are centered at zero in the full model. **Figure** 2 presents the visual predictive check for the full model stratified by biomarker status. Most of the observed scores lie within the 5th and 95th percentiles of the simulations; the 5th, 50th, and 95th percentiles of the observed data mostly fall within the 95% interpercentile range for the respective quantities in the simulations. The parametric bootstrap of the full model showed minimal bias for the parameter estimates, and the SEs were like those of the original model. The R code and output summary for the parametric bootstrap can be found in the **Supplementary Material**.

**Figure 3:**
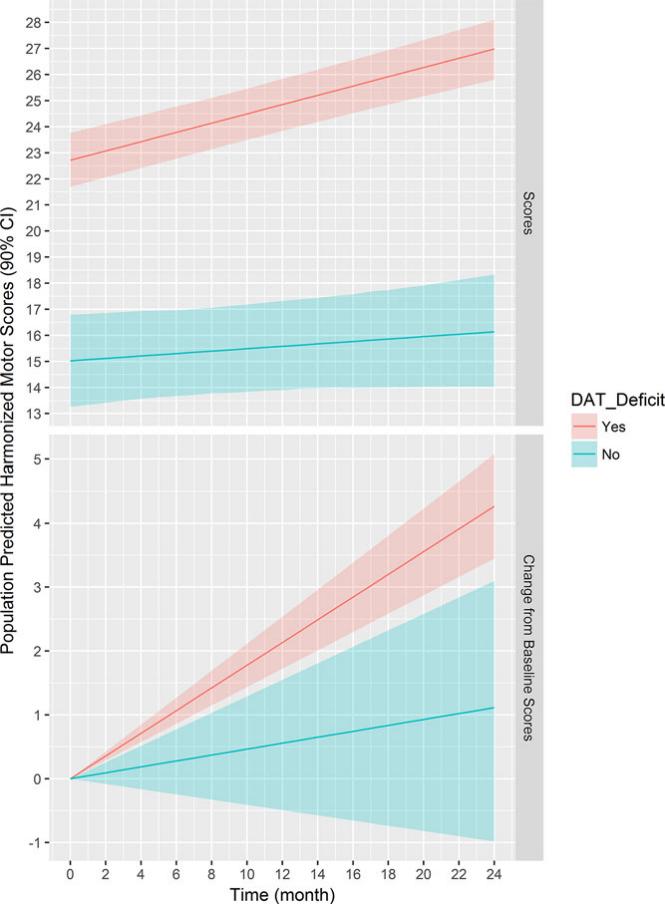
Population predicted harmonized motor scores. Shaded area is the 90% confidence interval (CI) from bootstrap. Predictions are for a Parkinson Research Examination of CEP‐1347 Trial (PRECEPT)‐like study with average age of 60 years old. DAT, dopamine transporter.

The population predicted harmonized motor scores over time are presented in **Figure** 3. The parameter estimates for the full model with their 90% CI from the bootstrap are presented in **Table** 2. As such, the following findings are noteworthy:

- The estimated effect of SWEDD on progression rate was ‐0.13 (90% CI = −0.23 to −0.04) point/month (one‐tailed *P* value = 0.01). This means that SWEDDs have an average monthly progression in the harmonized motor scores that is 0.05 (90% CI = −0.04 to 0.13) point/month or 0.13 point/month lower than those with DAT deficit (0.18 point/month; 90% CI = 0.14–0.21).
- The estimated effect of SWEDD on baseline was −7.69 (90% CI = −9.4 to −6.04) points; hence, SWEDDs have an average baseline harmonized motor score that is 7.69 points lower than those with DAT deficit.
- The estimated effect of year of age on baseline was 0.19 (90% CI = 0.14–0.24) points, which means that, on average, the baseline harmonized motor score increases by 0.19 points for each year of age. Thus, the baseline score for a typical 60‐year‐old subject with DAT deficit is expected to be 21.54 points.

**Table 2:** Parameter estimates from full model with 90% confidence intervals from bootstrap

**Parameter**	**Estimate**	**CI**
Intercept or baseline, points	10.08	6.83–13.61
Effect of PRECEPT study on baseline	1.20	0.01–2.34
Effect of year of age on baseline	0.19	0.14–0.24
Effect of SWEDD on baseline	−7.69	−9.4 to −6.04
Slope or progression rate (point/month)	0.18	0.14–0.21
Effect of SWEDD on progression rate	−0.13	−0.23 to –0.04
Variance of individual random effects for baseline (interindividual variability in baseline)	73.36	65.63–81.35
Variance of individual random effects for progression rate (interindividual variability in progression rate)	0.16	0.13–0.18
SD of the error distribution (points)	4.72	4.63–4.81

CI, confidence interval; DAT, dopamine transporter; SD, standard deviation; SWEDD, subjects without evidence of DAT deficit.

### Magnitude of motor scores worsening between biomarker statuses

The magnitude of motor scores worsening (i.e., change from baseline at 24 months) in DAT‐deficit and SWEDD subjects was 4.28 (90% CI = 3.45–5.08) and 1.12 (90% CI = −0.98 to 3.1) points, respectively. The average difference in the change from baseline score at 24 months between biomarker statuses was ‐3.16 (90% CI = −0.96 to −5.42) points, indicating that subjects with DAT deficit have an average of 3.16 points higher (worse) change‐from‐baseline motor score than SWEDDs.

### Subjects who experience a clinically important worsening of the motor scores

**Figure 4:**
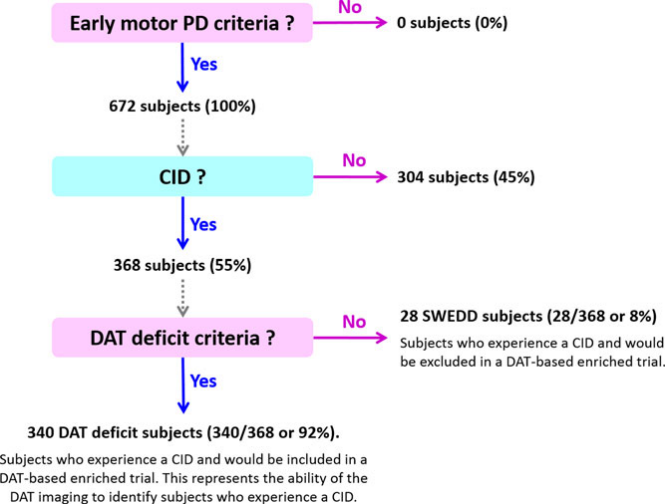
Ability of dopamine transporter **(**DAT) imaging to identify subjects who experience a clinically important worsening of the harmonized motor scores. Clinically important worsening or clinically important difference (CID) was defined as change from baseline in the harmonized motor scores of at least 3 points at 24 months. DAT‐based enriched trial is one that includes only DAT‐deficit subjects. Solid arrows mean that criteria are being applied. PD, Parkinson disease; SWEDD, subjects without evidence of DAT deficit.

The predicted individual change from baseline difference in the harmonized motor scores at 24 months was used to determine the subjects with a CID (i.e., difference ≥3 points). Of the 672 subjects diagnosed with early‐stage PD in the analysis data set, 368 were estimated to experience a CID. Of the 368 CID subjects, 340 had DAT deficit and 28 were SWEDDs. This means that the ability of the DAT imaging to identify subjects who experience a CID is 92.39%. Conversely, of the 368 CID subjects, 7.61% would be excluded in a DAT‐based enriched trial enrolling only DAT‐deficit subjects. Moreover, of the 304 non‐CID subjects, 243 had DAT deficit and 61 were SWEDDs. Hence, of the 89 total SWEDD subjects, 28 (31.46%) experienced a CID and 61 (68.54%) did not experience a CID. These results are summarized in **Figure** 4.

### Clinical trial simulations and statistical power

**Figure 5:**
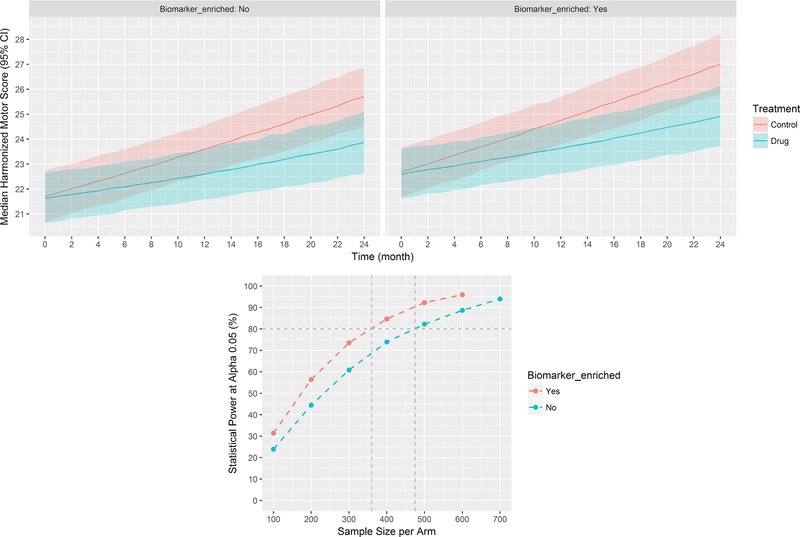
Simulated placebo‐controlled dopamine transporter **(**DAT) imaging enriched and nonenriched clinical trials with a drug effect of 50% reduction in the progression rate (*N* = 2,000 simulations). (**a**) Six hundred subjects per arm, shaded area is the 95% interpercentile range (confidence interval [CI]) for the collection of median scores from the simulations. (**b**) Statistical power vs. sample size.

From the 2,000‐simulated enriched and nonenriched placebo‐controlled clinical trials, the median harmonized motor scores over time for a 600‐subject per arm trial size is presented in **Figure** 5 **a**. The estimated power vs. sample size graph for DAT imaging enriched (i.e., only subjects with DAT deficit) and nonenriched (i.e., 15% of SWEDD subjects) trials is presented in **Figure** 5 **b**. Based on the simulations, interpolation shows that ∼475 subjects per arm would be required in a nonenriched placebo‐controlled clinical trial in order to detect a drug effect of 50% reduction in the progression rate with a 80% probability (type II error or β= 0.20^15^) at α= 0.05. Conversely, the same 80% probability of detecting an analogous drug effect at α= 0.05 is achieved with ∼355‐subjects/arm in an enriched clinical trial (an ∼24% reduction in sample size). Because the clinical trial simulations were performed to estimate the relative statistical power vs. sample size in trials with and without DAT imaging enrichment, dropout was not accounted for in that no statistically significant association between dropout and biomarker status was found (please refer to the Dropout Model section).

## DISCUSSION

In early PD therapeutic trials, ∼10–15% of subjects are identified as SWEDD.^4^ Previous longitudinal follow‐up of SWEDD subjects suggested minimal or absent clinical or imaging PD progression.^5^, ^16^ The objective of this work was to build upon previous findings and evaluate DAT neuroimaging as an enrichment biomarker in clinical trials targeting patients with early‐stage PD. Individual longitudinal data of subjects diagnosed with early‐stage PD in the PPMI PD and SWEDD cohorts, and in the PRECEPT control arm were analyzed. These data had a total of 672 patients with PD and a total of 4,521 observations in the baseline‐to‐25‐month interval. Individual baseline demographics and clinical characteristics were similar in both studies (**Table** 1). The dependent variable was the harmonized motor scores. The proportion of SWEDDs in the analysis data set was 13% and 14% for PPMI and PRECEPT, respectively. This also represents those patients potentially ineligible given a DAT‐based enrichment strategy. This, added to the percentage of ineligible screened patients due to other reasons (e.g., ∼11% in PRECEPT^10^), results in an estimated screen failure rate of about 25%. Not surprisingly, an increased screen failure rate is a feature of any such enrichment strategy,^17^ and should be considered by the sponsor in the context of overall trial execution time and cost.

DAT imaging could identify subjects with steeper motor scores worsening, allowing trial enrichment, and reduction of sample size. The rate of worsening in the motor scores between SWEDD and DAT‐deficit subjects was statistically and clinically significant different. SWEDD subjects have an average monthly progression in the harmonized score of 0.05 (90% CI = ‐0.04 to 0.13) point/month or 0.13 point/month lower, or less than half (one‐tailed *P* value = 0.01) than that in subjects with DAT deficit (0.18 point/month; 90% CI = 0.14–0.21). The effect of study on the progression rate was not statistically significant, suggesting that the rate of motor scores worsening of DAT‐deficit and SWEDD subjects are comparable between PPMI and PRECEPT. Subjects with DAT deficit have an average of 3.16 points higher (worse) change from baseline score at 24 months than SWEDDs, which is greater than the minimal CID of 3 points. Approximately 92% of the subjects estimated to experience a CID at 24 months were classified as DAT‐deficient subjects, meaning that most of the patients with a steeper clinical trajectory would be included in a DAT‐based enriched trial. To detect a drug effect of 50% reduction in the progression rate with an 80% probability (β = 0.20) at α = 0.05, a DAT‐based enrichment strategy was estimated to allow ∼24% reduction of trial size. A DAT‐based enrichment strategy, in which only DAT‐deficit subjects are enrolled in clinical trials of early PD, yields a more homogeneous and consistent worsening of the motor scores. Therefore, trials with a reduced sample size can be run to achieve the desired probability of detecting a drug effect.

Noteworthy findings of this work include: (a) confirmation that SWEDD subjects have a lower rate of motor scores worsening than those with DAT deficit in an integrated data set from two large clinical studies; (b) calculation of the average rate of worsening of motor scores over time in DAT‐deficit and SWEDD subjects by using the full longitudinal available data (besides only baseline and last study visit); (c) calculation of additional parameters from the mixed‐effects model that can differentiate sources of variability (e.g., individual variations vs. measurement noise) and be used to perform clinical trial simulations to guide future trial designs; and (d) demonstration of increased statistical power in trials with DAT imaging enrichment via Monte Carlo‐based clinical trial simulations.

Yet to be evaluated are: (a) nonlinear progression rate of the harmonized motor scores in broader PD stages; (b) additional covariates associated to progression rate; (c) beta‐distributed residual variability; (d) model predictive performance through external validation; and (e) effect of enrichment on trial duration, designs, and execution time and cost. Currently, an ongoing CPP consortium project aims to address such issues, envisioning a disease progression model‐based clinical trial enrichment tool. With additional studies at broader stages of PD,^9^ and knowledge from previously published UPDRS longitudinal models,^18^ CPP will investigate nonlinear models of various levels of complexity to describe the time course of motor scores. The present analysis focused on early motor PD, and the linear progression of the motor scores was adequate for this population given the results of model diagnostics and performance. Based on prior knowledge, characterizing the effect of baseline severity, genetic variants (e.g., glucocerebrosidase) and concomitant PD medications, for instance, in the intrinsic rate of disease progression may further inform patient selection in PD trials. Residual variability would be more correctly assumed to be beta‐distributed, given the bounded nature of motor scores. Although not evident in the present data set, the scores are subjected to ceiling and floor effects, which cause the residual variability to be heteroscedastic, with the variance approaching zero when the mean is close to the boundaries of the scale.^19^, ^20^ Further work can also evaluate different trial durations and designs; for instance, the possibility of a 1:2 placebo:treatment design in a Bayesian framework that leverages the knowledge gained by this model. Last, a cost analysis of DAT‐enrichment, comparing the saving from a reduced sample size vs. the DAT imaging cost and increased screen failures will inform the overall impact of DAT enrichment on trial costs.

In conclusion, the analysis of integrated data from an observational study and a randomized clinical trial shows that SWEDD subjects have a lower rate of progression of motor scores as compared to those subjects with DAT deficiency at baseline. Such finding helps to guide future clinical trials in that exclusion of SWEDD subjects (i.e., enrichment based on DAT imaging) will improve the chance of determining clinical benefit of drug candidates against PD at a reduced trial size, and prevent exposure to experimental treatments of patients who are unlikely to experience disease progression.

## Conflict of Interest

The authors declared no conflict of interest.

## Supporting information

Supporting Information
