# Supplementary material for: Dopamine Transporter Neuroimaging as an Enrichment Biomarker in Early Parkinson's Disease Clinical Trials: A Disease Progression Modeling Analysis
Source: Clin Transl Sci. 2017 Jul 27;11(1):63–70. doi: 10.1111/cts.12492 (PMC5759747; doi:10.1111/cts.12492)
Supplement: Supplementary file 1 — Supporting Information [file CTS-11-63-s001.docx]

**SUPPLEMENTARY MATERIAL**

**Dopamine transporter neuroimaging as an enrichment biomarker in early Parkinson’s disease clinical trials: a disease progression modeling analysis**

Daniela J. Conrado^1,*^, Timothy Nicholas^2^, Kuenhi Tsai^3^, Sreeraj Macha^3^, Vikram Sinha^3^, Julie Stone^3^, Brian Corrigan^2^, Massimo Bani^4^, Pierandrea Muglia^4^, Ian A. Watson^5^, Volker D. Kern^1^, Elena Sheveleva^1,6^, Kenneth Marek^7^, Diane T. Stephenson^1^ and Klaus Romero^1^ on behalf of the CPP Parkinson’s Disease Modeling and Simulation Working Group

*^1^Critical Path Institute, Tucson, AZ, USA; ^2^Pfizer Inc, Groton, CT, USA; ^3^Merck Sharp & Dohme, North Wales, PA, USA; ^4^UCB, Brussels, Belgium; ^5^Eli Lilly, Indianapolis, IN, USA; ^6^University of Arizona, Tucson, AZ, USA; ^7^Institute for Neurodegenerative Disorders, New Haven, CT, USA.*

***Corresponding author:**

Daniela J Conrado

Quantitative Medicine, Critical Path Institute

1730 E River Rd # 200, Tucson, AZ 85718

[DConrado@c-path.org](mailto:DConrado@c-path.org)

**Statistics and Modeling Methods**

The rate of progression on the harmonized motor scores was compared between SWEDD and DAT deficit subjects using a generalized linear mixed-effects model [1]. An unstructured covariance matrix was estimated. The calculated probabilities (*P*-values) were generated via F-testing based on the Kenward and Roger approach [2]. The following null and alternative hypotheses were tested at one-tailed $\alpha$ of 0.05:

- Null hypothesis ($H_{0}$): The fixed effect of interaction between biomarker status and time is equal to or greater than zero (i.e., the SWEDDs progression rate is equal to or greater than that of DAT deficit subjects).
- Alternative hypothesis ($H_{a}$): The fixed effect of interaction between biomarker status and time is less than zero (i.e., the SWEDDs progression rate is less than that of DAT deficit subjects).

All analyses used the lme4 package version 1.1-12 [1] and the pbkrtest package version 0.4-6 [2] in R version 3.3.1 [3].

*Fixed and Random Effects*

Pre-specified fixed effects were study, time, biomarker status, and interaction between biomarker status and time. The study effect was pre-specified to account for baseline motor scores differences between the PPMI and PRECEPT populations. The pre-specified mixed-effects model is represented in Equation S1 (i.e., full model). For comparison, a model without any adjustment for biomarker status was also fitted (i.e., reduced model). The fixed effect of interaction between study, and biomarker status and time was explored to compare progression rates between studies. Given the neurodegenerative nature of PD, the fixed effect of age was also explored. Pre-specified random effects were subject within study and measurement error. The random effect of subject within study was incorporated in intercept and time.

| $Y_{ijkl}=\mu+\alpha_{i}+b_{ij}+\gamma_{k}+\delta_{l}+f_{ij}+(\gamma\delta)_{kl}+e_{ijkl}$ | (S1) |
| --- | --- |

Where:

- $Y_{ijkl}$ is the harmonized motor score at time $l$ in subject $j$ with biomarker status $k$ in study $i$.
- $\mu$ is the intercept.
- $\alpha_{i}$ is the fixed effect of study $i$.
- $b_{ij}$ is the random effect of subject $j$ within study $i$ in intercept, where $b_{ij}$ ~ iid N(0, $\omega_{intercept}^{2}$).
- $\gamma_{k}$ is the fixed effect of biomarker status $k$.
- $\delta_{l}$ is the fixed effect of continuous time $l$.
- $f_{ij}$ is the random effect of subject $j$ within study $i$ in time, where $f_{ij}$ ~ iid N(0, $\omega_{time}^{2}$).
- $(\gamma\delta)_{kl}$ is fixed effect of interaction between biomarker status $k$ and time $l$.
- $e_{ijkl}$ is the random effect of measurement at time $l$ in subject $j$ with biomarker status $k$ in study $i$.

*Model Selection Criteria and Performance*

Model selection was guided by the Akaike information criterion (AIC) with a per-parameter penalty of 2. Model diagnostic graphs were generated to evaluate the extent to which the predicted and observed harmonized motor scores matched (i.e., goodness-of-fit), and to evaluate the normality assumption. These were (a) individual-observed *versus* individual-predicted harmonized motor scores; (b) individual-predicted harmonized motor scores *versus* Pearson-residuals; (c) time *versus* Pearson-residuals; (d) histogram of residuals; (d) theoretical quantiles *versus* sample quantiles of residuals; (e) individual's observed and predicted harmonized motor scores *versus* time; (f) box plots of individual random effects for rate of progression stratified by DAT biomarker status.

With the final model, a parametric bootstrap (2000 simulations) was performed to determine the magnitude of difference between the mean parameter estimates from the original model and those from the bootstrap (i.e., bias). In addition, the standard errors of the parameters from the original model were compared to those from the bootstrap, and the 90% confidence intervals were calculated from the bootstrap results.

To evaluate model performance, 1000 datasets identical in structure and covariate values to the original were simulated, using the parameter estimates from the final model. The time courses of the harmonized motor scores from the simulations were generated as visual predictive checks, (VPC) at each selected percentile (5^th^, 50^th^, 95^th^).

**References**

[1] Douglas Bates, Martin Mächler, Ben Bolker, and Steve Walker, “Fitting Linear Mixed-Effects Models Using lme4 | Bates | Journal of Statistical Software.” [Online]. Available: https://www.jstatsoft.org/article/view/v067i01. [Accessed: 06-Jan-2017].

[2] Ulrich Halekoh and Søren Højsgaard, “A Kenward-Roger Approximation and Parametric Bootstrap Methods for Tests in Linear Mixed Models  The R Package pbkrtest | Halekoh | Journal of Statistical Software.” [Online]. Available: https://www.jstatsoft.org/article/view/v059i09. [Accessed: 06-Jan-2017].

[3] R Core Team, *R: A language and environment for statistical computing. R Foundation for Statistical Computing*. Vienna, Austria, 2016.

**Reduced Model**

reducedModel <- **lmer**(HarmonizedScore ~ Study + TimeMonth + Age +
 (1 + TimeMonth | UniqueSubjectId), data = myData)
**summary**(reducedModel)

## Linear mixed model fit by REML t-tests use Satterthwaite approximations
## to degrees of freedom [lmerMod]
## Formula: HarmonizedScore ~ Study + TimeMonth + Age + (1 + TimeMonth |
## UniqueSubjectId)
## Data: myData
##
## REML criterion at convergence: 29707.2
##
## Scaled residuals:
## Min 1Q Median 3Q Max
## -3.8294 -0.5065 -0.0421 0.4528 4.9974
##
## Random effects:
## Groups Name Variance Std.Dev. Corr
## UniqueSubjectId (Intercept) 80.0187 8.945
## TimeMonth 0.1569 0.396 -0.23
## Residual 22.2989 4.722
## Number of obs: 4521, groups: UniqueSubjectId, 672
##
## Fixed effects:
## Estimate Std. Error df t value Pr(>|t|)
## (Intercept) 8.97053 2.14568 669.40000 4.181 3.29e-05 ***
## StudyPRECEPT 1.17401 0.76653 654.10000 1.532 0.126
## TimeMonth 0.16097 0.01918 597.10000 8.393 4.44e-16 ***
## Age 0.19265 0.03442 664.40000 5.597 3.20e-08 ***
## ---
## Signif. codes: 0 '***' 0.001 '**' 0.01 '*' 0.05 '.' 0.1 ' ' 1
##
## Correlation of Fixed Effects:
## (Intr) SPRECE TmMnth
## StdyPRECEPT -0.187
## TimeMonth -0.059 0.012
## Age -0.980 0.085 0.001

*# Type 3 anova table with additional F statistics and*
*# denominator degrees of freedom*
*# calculated based on Kenward-Roger's approximation*
if(**require**(pbkrtest))
 **anova**(reducedModel, ddf = "Kenward-Roger", type=3)

## Note: method with signature 'sparseMatrix#ANY' chosen for function 'kronecker',
## target signature 'dgCMatrix#ngCMatrix'.
## "ANY#sparseMatrix" would also be valid

## Analysis of Variance Table of type III with Kenward-Roger
## approximation for degrees of freedom
## Sum Sq Mean Sq NumDF DenDF F.value Pr(>F)
## Study 52.25 52.25 1 700.68 2.343 0.1263
## TimeMonth 1570.39 1570.39 1 694.24 70.425 2.675e-16 ***
## Age 697.72 697.72 1 707.49 31.290 3.178e-08 ***
## ---
## Signif. codes: 0 '***' 0.001 '**' 0.01 '*' 0.05 '.' 0.1 ' ' 1

**Full Model**

fullModel <- **lmer**(HarmonizedScore ~ Study + TimeMonth + Age +
 DatDeficit + TimeMonth*DatDeficit +
 (1 + TimeMonth | UniqueSubjectId), data = myData)
**summary**(fullModel)

## Linear mixed model fit by REML t-tests use Satterthwaite approximations
## to degrees of freedom [lmerMod]
## Formula:
## HarmonizedScore ~ Study + TimeMonth + Age + DatDeficit + TimeMonth *
## DatDeficit + (1 + TimeMonth | UniqueSubjectId)
## Data: myData
##
## REML criterion at convergence: 29629.6
##
## Scaled residuals:
## Min 1Q Median 3Q Max
## -3.8184 -0.4997 -0.0401 0.4508 4.9856
##
## Random effects:
## Groups Name Variance Std.Dev. Corr
## UniqueSubjectId (Intercept) 73.3550 8.5648
## TimeMonth 0.1551 0.3938 -0.27
## Residual 22.2966 4.7219
## Number of obs: 4521, groups: UniqueSubjectId, 672
##
## Fixed effects:
## Estimate Std. Error df t value Pr(>|t|)
## (Intercept) 10.08268 2.03864 670.80000 4.946 9.60e-07 ***
## StudyPRECEPT 1.19960 0.72579 653.30000 1.653 0.0988 .
## TimeMonth 0.17840 0.02049 595.10000 8.707 < 2e-16 ***
## Age 0.19096 0.03260 663.70000 5.858 7.37e-09 ***
## DatDeficitNo -7.69389 1.04638 671.70000 -7.353 5.66e-13 ***
## TimeMonth:DatDeficitNo -0.13185 0.05662 606.60000 -2.329 0.0202 *
## ---
## Signif. codes: 0 '***' 0.001 '**' 0.01 '*' 0.05 '.' 0.1 ' ' 1
##
## Correlation of Fixed Effects:
## (Intr) SPRECE TmMnth Age DtDfcN
## StdyPRECEPT -0.186
## TimeMonth -0.071 0.008
## Age -0.977 0.085 0.001
## DatDeficitN -0.076 -0.007 0.136 0.009
## TmMnth:DtDN 0.026 0.000 -0.362 0.000 -0.377

*# Type 3 anova table with additional F statistics and*
*# denominator degrees of freedom*
*# calculated based on Kenward-Roger's approximation*
if(**require**(pbkrtest))
 **anova**(fullModel, ddf = "Kenward-Roger", type=3)

## Analysis of Variance Table of type III with Kenward-Roger
## approximation for degrees of freedom
## Sum Sq Mean Sq NumDF DenDF F.value Pr(>F)
## Study 60.84 60.84 1 689.24 2.729 0.09902 .
## TimeMonth 351.85 351.85 1 695.04 15.781 7.855e-05 ***
## Age 764.37 764.37 1 697.31 34.282 7.342e-09 ***
## DatDeficit 1205.40 1205.40 1 935.93 54.062 4.237e-13 ***
## TimeMonth:DatDeficit 120.90 120.90 1 694.78 5.422 0.02017 *
## ---
## Signif. codes: 0 '***' 0.001 '**' 0.01 '*' 0.05 '.' 0.1 ' ' 1

**Models Comparison**

if(**require**(pbkrtest))
**anova**(reducedModel, fullModel)

## refitting model(s) with ML (instead of REML)

## Data: myData
## Models:
## object: HarmonizedScore ~ Study + TimeMonth + Age + (1 + TimeMonth |
## object: UniqueSubjectId)
## ..1: HarmonizedScore ~ Study + TimeMonth + Age + DatDeficit + TimeMonth *
## ..1: DatDeficit + (1 + TimeMonth | UniqueSubjectId)
## Df AIC BIC logLik deviance Chisq Chi Df Pr(>Chisq)
## object 8 29713 29765 -14849 29697
## ..1 10 29637 29701 -14809 29617 80.043 2 < 2.2e-16 ***
## ---
## Signif. codes: 0 '***' 0.001 '**' 0.01 '*' 0.05 '.' 0.1 ' ' 1

**Parametric Bootstrap**

mySummary <- function(.){
 **c**(FIXED=**fixef**(.),
 RANDOM.UniqueSubjectId.variance.Intercept=
 **as.data.frame**(**VarCorr**(., comp="Variance"))[1, "vcov"],
 RANDOM.UniqueSubjectId.variance.TimeMonth=
 **as.data.frame**(**VarCorr**(., comp="Variance"))[2, "vcov"],
 RANDOM.UniqueSubjectId.covariance.Intercept.TimeMonth=
 **as.data.frame**(**VarCorr**(., comp="Variance"))[3, "vcov"],
 RANDOM.Error.stddev=**sigma**(.))
}

boot <-
 **bootMer**(fullModel, mySummary, nsim = 2000, seed = 123,
 type = "parametric", use.u = F)

boot

##
##
## Call:
## bootMer(x = fullModel, FUN = mySummary, nsim = 2000, seed = 123,
## use.u = F, type = "parametric")
##
##
## Bootstrap Statistics :
## original bias std. error
## t1* 10.0826753 -0.0297680515 2.04567275
## t2* 1.1995965 -0.0245763392 0.71510227
## t3* 0.1783988 -0.0006160167 0.02061307
## t4* 0.1909576 0.0005177579 0.03251828
## t5* -7.6938873 0.0008908005 1.02749878
## t6* -0.1318539 0.0004591157 0.05630850
## t7* 73.3550395 0.0311083467 4.69893163
## t8* 0.1551090 0.0006421777 0.01347240
## t9* -0.9241082 -0.0055801200 0.18444206
## t10* 4.7219229 -0.0003985295 0.05617601

*# ci*
boot_values <- (**as.data.frame**(boot))
parameter_ci <- **data.frame**()
for (myIndex in 1:**ncol**(boot_values)){
 tmp <- **data.frame**(INDEX=myIndex, LowerCI=NA, UpperCI=NA)
 tmp$LowerCI <-
 (**boot.ci**(boot, conf = 0.90, index=myIndex, type="perc"))$percent[,4]
 tmp$UpperCI <-
 (**boot.ci**(boot, conf = 0.90, index=myIndex, type="perc"))$percent[,5]
 parameter_ci <- **rbind**(parameter_ci, tmp)
}
parameter_ci$Parameter <- **names**(boot_values)
parameter_ci <- parameter_ci[, **c**("Parameter", "LowerCI", "UpperCI")]
parameter_ci

## Parameter LowerCI
## 1 FIXED.(Intercept) 6.826621452
## 2 FIXED.StudyPRECEPT 0.009840659
## 3 FIXED.TimeMonth 0.143596985
## 4 FIXED.Age 0.136046365
## 5 FIXED.DatDeficitNo -9.398540433
## 6 FIXED.TimeMonth:DatDeficitNo -0.225655058
## 7 RANDOM.UniqueSubjectId.variance.Intercept 65.632710781
## 8 RANDOM.UniqueSubjectId.variance.TimeMonth 0.134761905
## 9 RANDOM.UniqueSubjectId.covariance.Intercept.TimeMonth -1.238880286
## 10 RANDOM.Error.stddev 4.630701825
## UpperCI
## 1 13.61337432
## 2 2.33552112
## 3 0.21160540
## 4 0.24203145
## 5 -6.04266225
## 6 -0.03999471
## 7 81.34985444
## 8 0.17906259
## 9 -0.63284620
## 10 4.81428929

Table S1. Visit-level data exclusions.

| Exclusion Reason | Number of Observations |
| --- | --- |
| Time equal to or greater than 25 months | 1959 |
| Missing harmonized motor score | 62 |
| Screening | 683 |

Table S2. Dropout model selection.

| Model | Number of Parameters (k) | Log likelihood (LL) | -2 Log Likelihood (-2LL) | Modified Akaike’s Information Criterion (AIC_mod_) |
| --- | --- | --- | --- | --- |
| **Base model** | | | | |
| **Gompertz** | **2** | **-292** | **585** | **592** |
| Exponential | 1 | -295 | 590 | 594 |
| Weibull | 2 | -294 | 587 | 595 |
| Log-logistic | 2 | -294 | 587 | 595 |
| Log-normal | 2 | -295 | 589 | 597 |
| Gamma | 3 | -293 | 587 | 598 |
| **Inclusion of covariates** | | | | |
| Gompertz + Age | 3 | -291 | 582 | 594 |
| Gompertz + Study | 3 | -292 | 583 | 595 |
| Gompertz + Biomarker Status | 3 | -292 | 584 | 595 |
| Gompertz + Baseline Harmonized Motor Score | 3 | -292 | 585 | 596 |

$${AIC}_{mod}=3.841\times k-2\mathrm{LL}$$

Figure S1. Schematic for PRECEPT and PPMI.

**SWEDD**

**Analyzed**

**n= 62**

**PD disease**

**Analyzed**

**n= 419**

**SWEDD**

**Analyzed**

**n=26**

**PD disease**

**Analyzed**

**n= 165**

**Safety and Efficacy Study of CEP-1347 in the Treatment of Parkinson's Disease (PRECEPT) -** NCT00040404

**Sponsor: Cephalon**

**Screened**

**Enrolled n=64**

Excluded 4 Excluded 2

**Enrolled/allocated**

Analyzed

**Consented n=82**

**Enrolled n=423**

**PD subjects SWEDD subjects**

**Consented n=488**

**THE PARKINSON’S PROGRESSION MARKERS INITIATIVE (PPMI)**

**Sponsor:** **The** **Michael J Fox Foundation**

**Screened for eligibility n=904**

**Total number of patients for allocation n=806**

**CEP-1347 50 mg BID n=198**

**Placebo**

**n=191**

**CEP-1347 25mg BID n=212**

**CEP-1347 10 mg BID n=205**

Figure S2. Diagnostic graphs of the full model.

| **(A) All data**  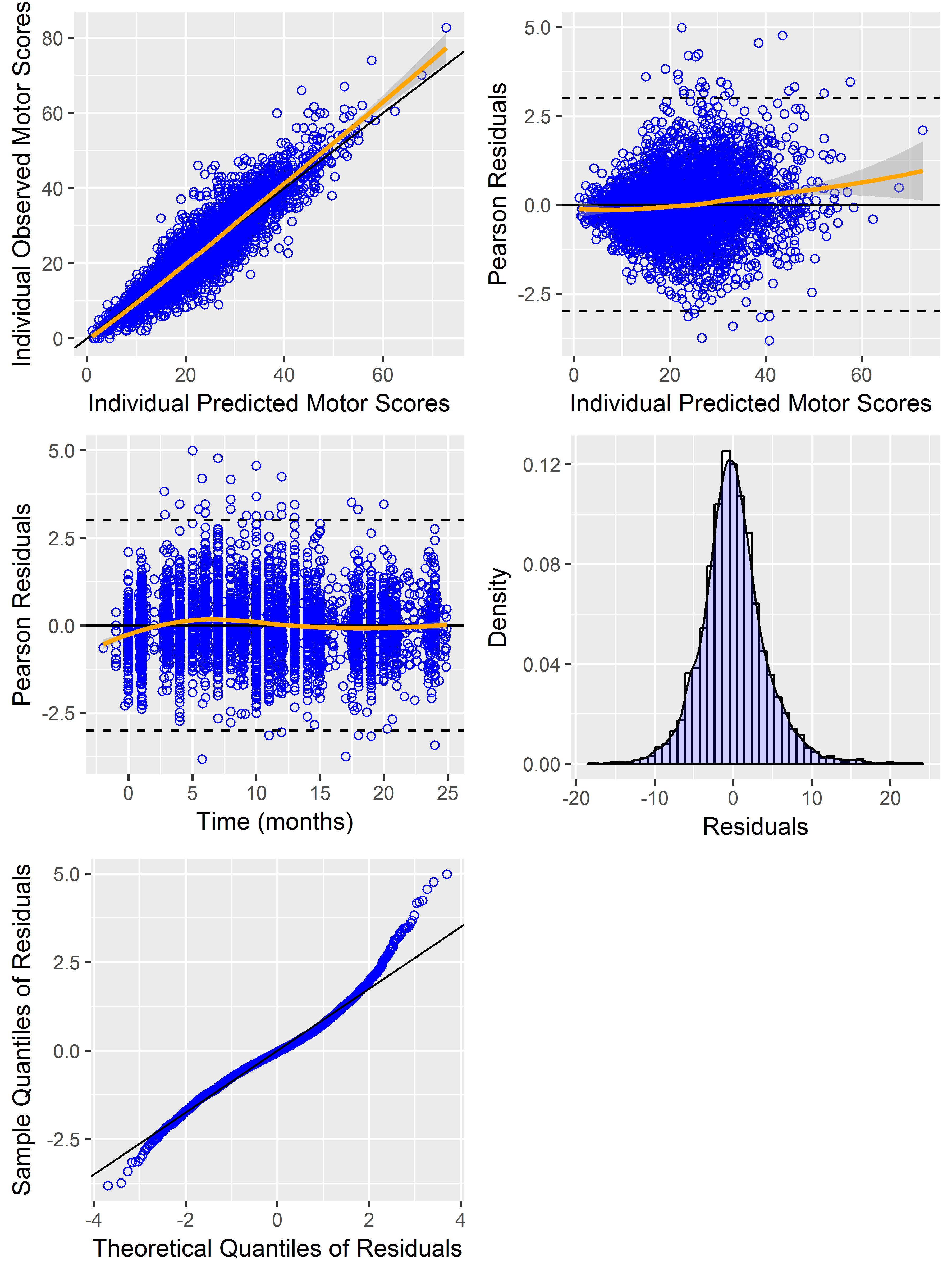 | **(B) Only SWEDD**  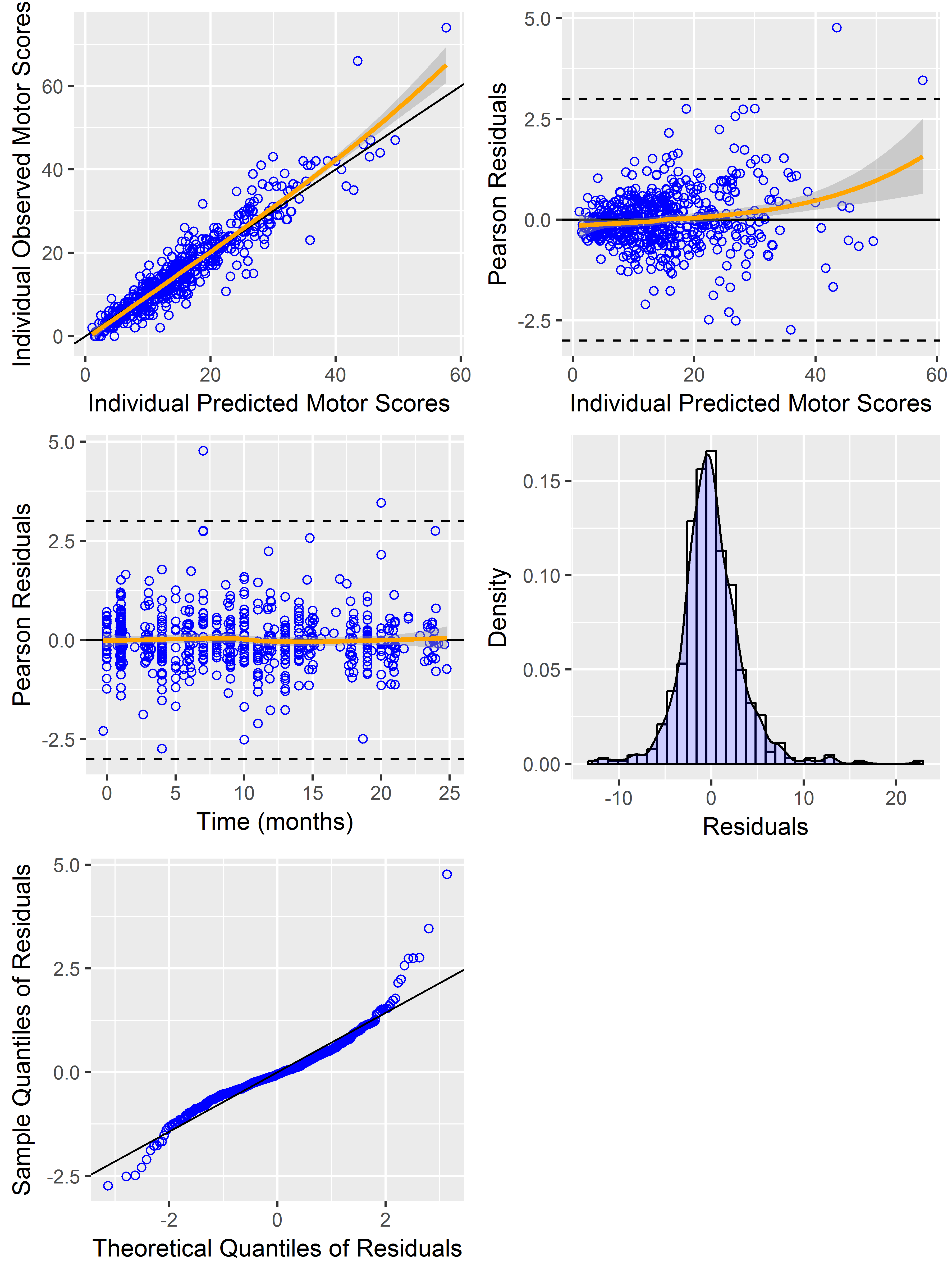 |
| --- | --- |
| η-shrinkage was 6% for baseline and 20% for progression rate. η-shrinkage was quantified as $1-SD(\eta_{EBE})/\omega$, where $\eta_{EBE}$ are the empirical Bayes estimates of the individual random effects, and $\omega$ is the standard deviation of the random effect distribution determined by the mixed-effects model. | |

Figure S3. Individuals observed (Obs) and predicted (IPred) harmonized motor scores *versus* time for the full model.

| **(A) DAT deficit (first 9 in the dataset)**  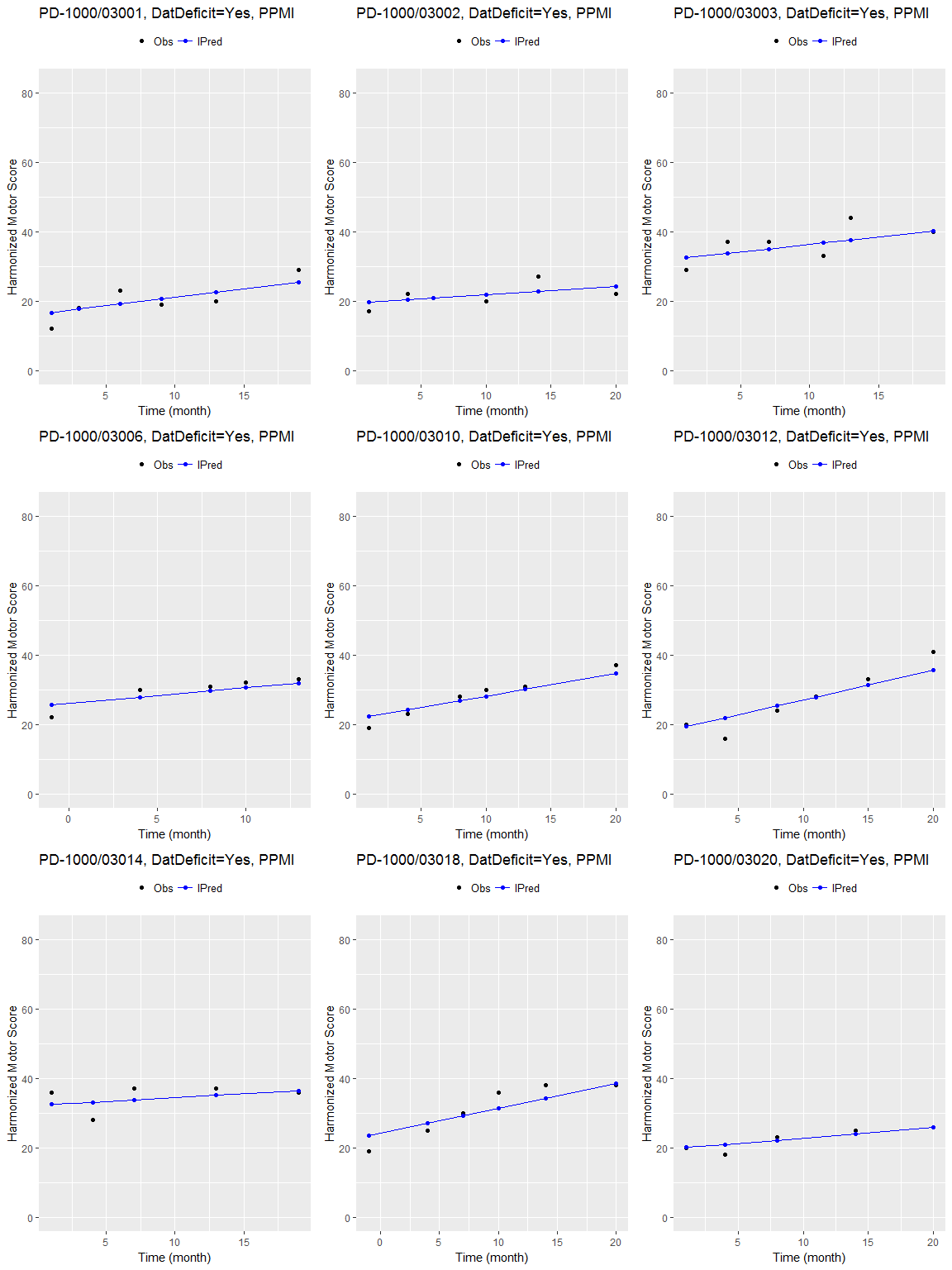 | **(B) SWEDD (first 9 in the dataset)**  **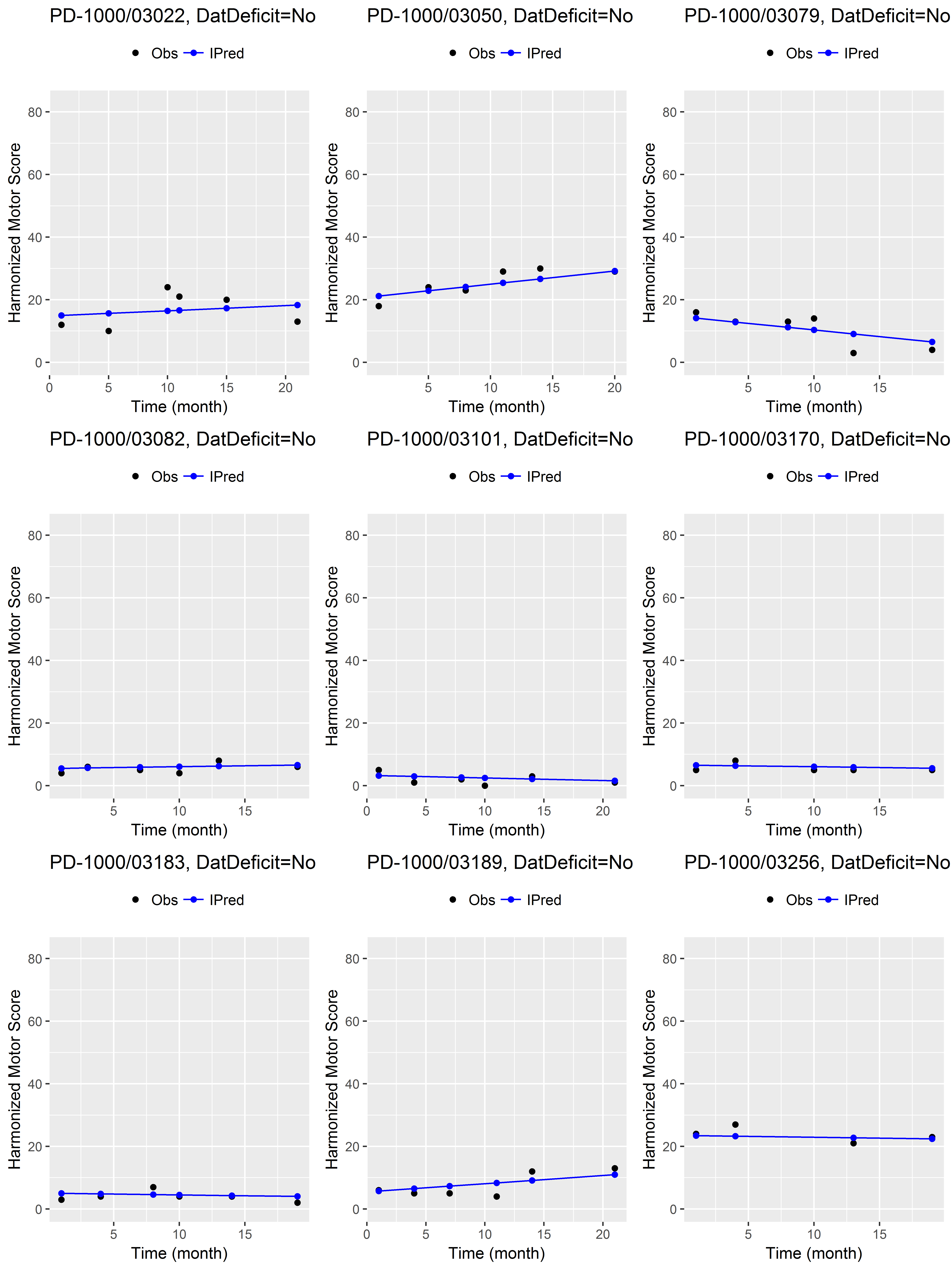** |
| --- | --- |

Figure S4. Individual random effects for rate of progression stratified by DAT biomarker status for the reduced and full model.

| 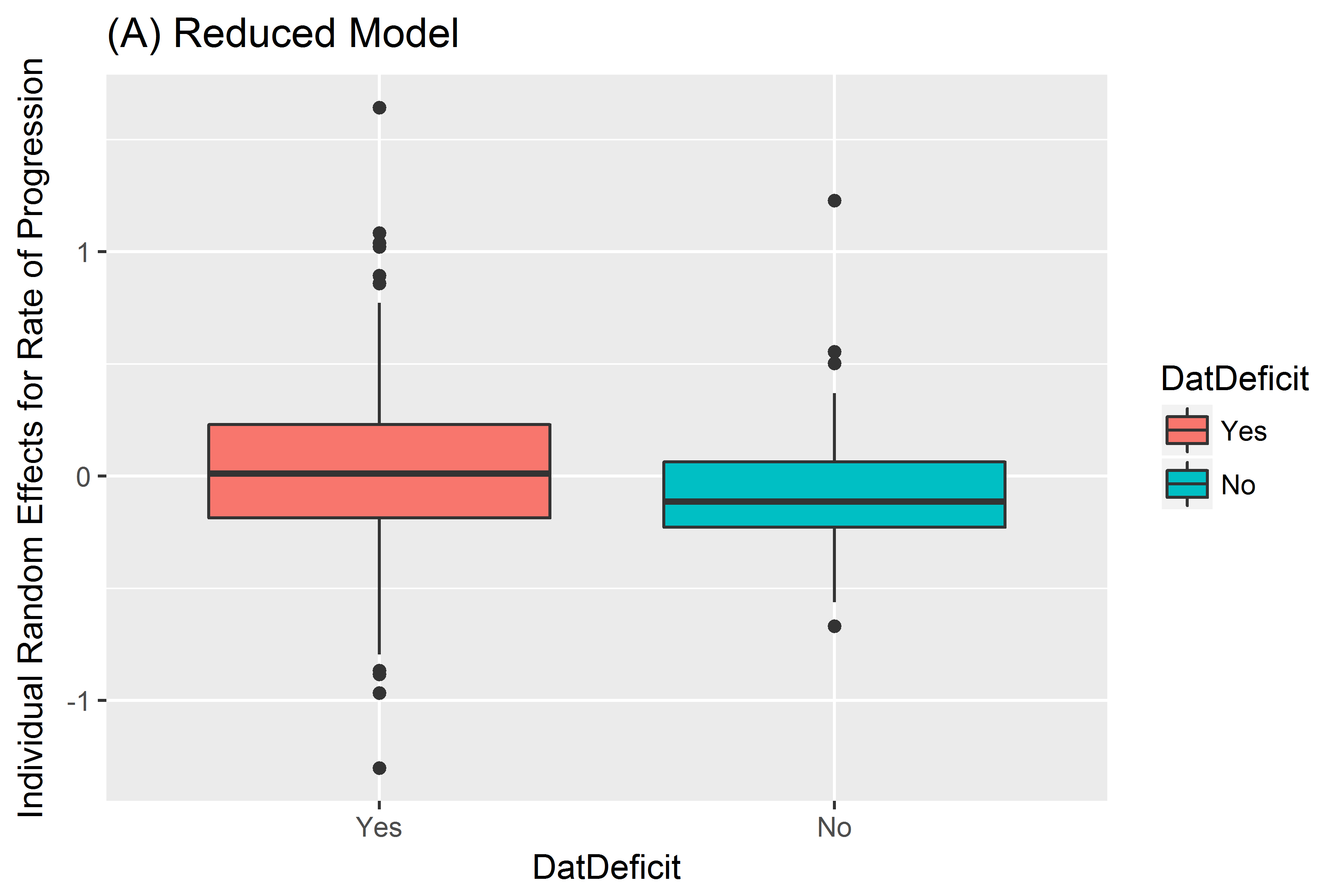 | 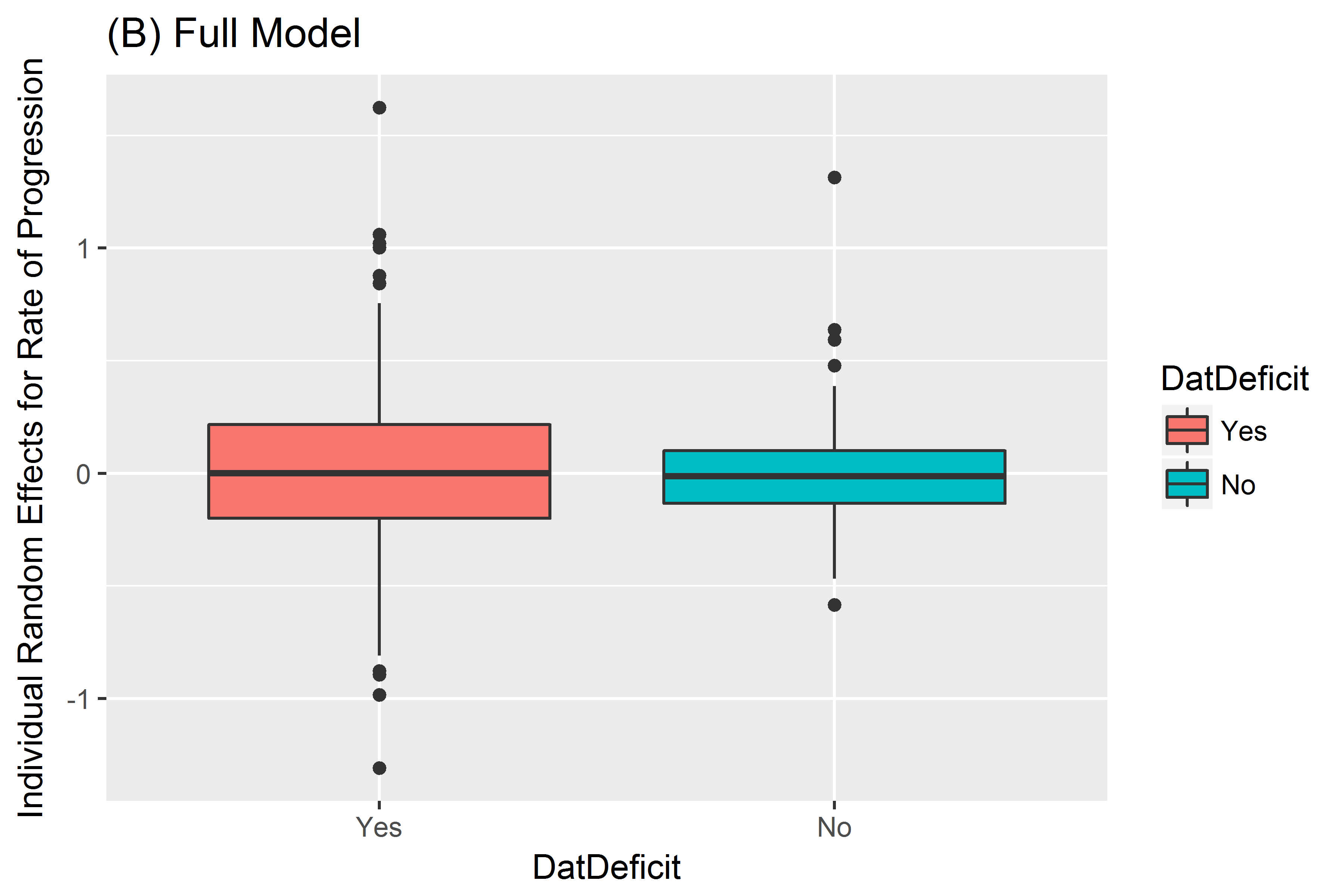 |
| --- | --- |
